# Commentary: Anterior Cingulate Cortex and Response Conflict: Effects of Frequency, Inhibition and Errors

**DOI:** 10.3389/fnbeh.2018.00171

**Published:** 2018-08-08

**Authors:** Sara Palermo, Mario Stanziano, Rosalba Morese

**Affiliations:** ^1^Department of Psychology, University of Turin, Turin, Italy; ^2^A.O.U. Citta della Salute e della Scienza di Torino, Turin, Italy; ^3^Faculty of Communication Sciences, Università della Svizzera Italiana, Lugano, Switzerland

**Keywords:** response inhibition paradigm, fMRI, go-nogo task, Low-frequency events, Parkinson's disease and related movement disorders, clinical investigations, neuropsychological data, Neurocognitive approach

Functional Magnetic Resonance Imaging (fMRI) is a relatively new imaging technique, which enables mapping of brain functions with high spatial resolution. In recent years, fMRI have had a huge impact on the study of brain function, both in healthy and diseased participants. In particular, event-related fMRI is used to investigate the neural correlates involved in numerous motor, cognitive, disinhibition and impulsivity tasks and to gain a better understanding about how normal neural functions are altered in disease (Palermo and Morese, 2018). Considering clinical diagnosis, fMRI methods are being applied to investigate neural correlates associated with patients' neuropsychological and neuropsychiatric symptomatology, since it is a powerful methodology to record the cerebral BOLD signal avoiding technical problems due to long acquisition time defined for block-design in clinical fMRI studies. This is particular important in the case of movement disorders (Palermo and Morese, 2018).

One of the most fruitful event-related paradigm to study executive function related to action-monitoring and response-inhibition in neurocognitive disorders and in neuropsychiatric diseases is the GO-NoGO task (Kiehl et al., 2000; Liddle et al., 2001). It involves the visual presentation of different stimuli with different frequencies in order to create conflicts in processing and in choosing between the response (GO) or no response (NoGO), i.e., response inhibition for NoGO trials. Different and complex versions of this paradigm—allowing the manipulation of stimulus-response associations—were created. Braver et al. (2001) used this task condition to investigate the role of anterior cingulate cortex (ACC) activity during response inhibition, response selection, and target detection tasks. They manipulated the levels of stimulus frequency to investigate whether ACC was most responsive in the case the NoGO event occurred with low frequency rates. The rational of their study was that the low-frequency responding might provide a minimal condition for eliciting such conflict between the response (GO) or no response (NoGO). They hypothesized that high levels of response conflict, during the processing of low-frequency events, were correlated with response inhibition for NoGO trials. In their study healthy participants were instructed to inhibit response to infrequent NoGO stimuli (the letter “X” = 17% frequency) and to respond to frequent GO stimuli (the “non-X” letters = 83% frequency). Their results confirmed the hypothesis that the ACC has a central role to detect processing conflict when low-frequency responses are being executed. Moreover, ACC showed heightened activity during response inhibition and on trials in which errors were committed. Their results are consistent with the hypothesis that the ACC serves as a generic detector of processing conflict with low-frequency responses, but also leave open the possibility that further clinical researches. This aspect is of particular importance, for example, in Parkinson's disease, in which the loss of dopaminergic neurons impacts on the functioning of two fronto-striatal circuits involved in different aspects of behavior: (i) the orbito-frontal cortex, associated with decision-making, impulse control, mood expression and perseveration; (ii) ACC, associated with conflict monitoring, intention, response initiation/inhibition (Zgaljardic et al., 2006; Palermo et al., 2017). In our experience (Palermo et al., 2017; Palermo and Morese, 2018), fMRI response-inhibition task is useful for better characterizing the clinical profile while evaluating treatment options. Interestingly, impaired response-inhibition is an example of the motor/behavioral aspect of impulse control. Its assessment is supposed to be particularly useful in the PD post-diagnostic phase, to better identify individuals at risk of developing response-inhibition disabilities with dopaminergic medication. Indeed, establishing a relationship between disinhibition and executive functions could potentially be helpful for therapeutic research and for the efficient targeting of symptom relief in PD. Theoretical models will be more effective if they integrate fMRI and neuropsychological data according to a neurocognitive approach to Parkinson's disease. In general, GO-NoGO fMRI paradigm is a valuable method to better understand patients' metacognitive-executive profile.

We would like to conclude by reiterating the importance of this type of task in the evaluation of patients, remembering that the response inhibition is worthy of clinical investigations in pathologies with different etiopathogenesis.

## Author contributions

All authors listed have made a substantial, direct and intellectual contribution to the work, and approved it for publication.

### Conflict of interest statement

The authors declare that the research was conducted in the absence of any commercial or financial relationships that could be construed as a potential conflict of interest.
